# Dietary calcium intake was related to the onset of pre‐eclampsia: The TMM BirThree Cohort Study

**DOI:** 10.1111/jch.14606

**Published:** 2022-12-28

**Authors:** Hisashi Ohseto, Mami Ishikuro, Taku Obara, Keiko Murakami, Tomomi Onuma, Aoi Noda, Ippei Takahashi, Fumiko Matsuzaki, Fumihiko Ueno, Noriyuki Iwama, Masahiro Kikuya, Hirohito Metoki, Junichi Sugawara, Shinichi Kuriyama

**Affiliations:** ^1^ Tohoku University Graduate School of Medicine Sendai Japan; ^2^ Tohoku Medical Megabank Organization Tohoku University Sendai Japan; ^3^ Tohoku University Hospital Sendai Japan; ^4^ Teikyo University School of Medicine Tokyo Japan; ^5^ Faculty of Medicine Tohoku Medical and Pharmaceutical University Sendai Japan; ^6^ International Research Institute of Disaster Science Tohoku University Sendai Japan

**Keywords:** antenatal care, calcium, cohort study, food frequency questionnaire, magnesium, potassium, prediction model, pre‐eclampsia, pre‐eclampsia superimposed on chronic hypertension, pregnancy

## Abstract

This study aimed to explore the relationship between dietary electrolyte intake and the prevalence of hypertensive disorders of pregnancy (HDP) subtypes. Our analysis included 19 914 pregnant women from the Tohoku Medical Megabank Project Birth and Three‐Generation Cohort Study. A food frequency questionnaire was used to estimate dietary calcium, potassium, sodium, and magnesium intakes. HDP was determined based on the medical records during regular antenatal care. Logistic regression analysis assessed the relationship between dietary electrolytes intake quintiles, and HDP subtypes with adjustment for basic characteristics. Dietary electrolyte intakes were applied for the prediction model. Of the cohort, 547 participants delivered with pre‐eclampsia (PE), 278 with superimposed PE (SP), and 896 with gestational hypertension (GH). PE was associated with low crude calcium intake (odds ratio of the first quintile [<251 mg/day] to the fifth quintile [>623 mg/day] and 95% confidence interval, 1.31 [1.00–1.70]) and *P* for trend was .02. SP was not associated with any nutritional intake; however, the combined outcome of PE and SP was related to low crude calcium and potassium and energy‐adjusted calcium, potassium, and magnesium intakes (*P* for trend, .01, .048, .02, .04, and .02, respectively). The same tendency was observed for GH. A prediction model that included crude calcium and potassium intakes performed better than a model without them. In conclusion, low dietary calcium, potassium, and magnesium were associated with higher HDP subtypes prevalence. The prediction model implied that crude calcium and potassium intakes might play a critical role in PE and SP pathogenesis.

## 1 INTRODUCTION

Pre‐eclampsia (PE) is characterized by hypertension and proteinuria after 20 weeks of gestation^1^, ^2^ and affects about 3.4% of all pregnant women.^3^ Women with PE are at an increased risk of morbidity^4^, ^5^ and future cardiovascular complications.^6^ Previous studies have suggested that women, especially those with risk factors for PE, could benefit from daily low‐dose aspirin.^2^, ^7^, ^8^ However, considering possible adverse effects and financial burden, a daily aspirin dose might not be suitable for every pregnant woman. Therefore, modifiable risks of PE for effective clinical practice in early pregnancy should be elucidated.

Nutrition can be a modifiable factor for PE. However, the evidence among studies is inconsistent. A meta‐analysis of 13 trials with 15 730 women suggested that calcium supplementation had a significant preventive effect on PE, especially among pregnant women with low baseline calcium intake.^9^ In contrast, a meta‐analysis from 2014 focusing on dietary calcium intake and based on unadjusted odds ratios in ten studies^,^ and adjusted odds ratios in three case‐control studies, found no significant relationship between dietary calcium intake and the onset of PE or hypertensive disorders of pregnancy (HDP).^10^ Furthermore, a cohort study from 2020 examining 33 894 pregnant women in Japan^11^ found no significant relationship between dietary calcium intake and HDP. Evidence on the relationship between PE and other electrolytes, including potassium, sodium, and magnesium, is lacking.^12^, ^13^, ^14^ Studies found insufficient evidence supporting the use of nutritional interventions other than electrolytes to reduce the risk of PE, including vitamins C and E,^15^ fish oil,^16^ garlic supplementation,^17^ vitamin D,^18^ and folic acid.^19^

Further research examining the relationship between nutritional intake and PE is needed to identify high‐risk pregnant women and develop an optimal clinical strategy. This study investigated the relationship between dietary nutritional intake, particularly calcium, and the prevalence of the HDP subtypes. We also investigated whether dietary nutritional intake could help develop an accurate prediction model.

## 2 METHODS

### 2.1 Study population

The Tohoku Medical Megabank Project Birth and Three‐Generation (TMM BirThree) Cohort Study^20^, ^21^ was initiated in July 2013 to collect in utero and subsequent exposure and outcome information to promote personalized healthcare and medicine. Approximately 50 obstetric clinics and hospitals in the Miyagi Prefecture, Japan, participated in recruiting 22 493 pregnant women. The present study excluded those who withdrew their consent (*n* = 335), had multiple pregnancies (*n* = 328), or had missing HDP diagnosis (*n* = 827) or food frequency questionnaire (FFQ) information (*n* = 1089). Therefore, this study analyzed 19,914 pregnant women. The ethics approval was obtained from the Ethics Committee of the Tohoku Medical Megabank Organization (2013‐1‐103‐1), and all participants provided written informed consent for study inclusion.

### 2.2 Exposures and other covariates

The participants completed the FFQ during their gestation period (mean ± SD, 140 ± 52 days) based on their eating habits during the year before the response date. We calculated dietary nutrition intake, including energy, protein, carbohydrate, fats, and electrolytes. The present study focused on calcium,^13^ and other electrolytes: potassium, sodium, magnesium, and sodium‐to‐potassium ratio (Na/K)^11^, ^12^, ^14^, ^22^, ^23^ with and without using the residual method to adjust for the total energy intake.

We selected verified or possible risk factors for HDP as covariates, including age during antenatal care at 10–13 weeks of gestation,^24^ pre‐pregnancy body mass index (BMI),^24^ chronic hypertension (CH), systemic lupus erythematosus (SLE),^24^ diabetes mellitus (DM),^24^ family (mother and sisters) history of HDP,^24^ conception by in vitro fertilization (IVF),^24^ parity (nulliparous, parous with previous HDP, or parous without previous HDP),^24^ educational attainment,^25^ smoking status during early pregnancy,^26^, ^27^ alcohol consumption during early pregnancy,^28^ and family (father, mother, brothers, and sisters) history of CH.^29^ Basic maternal characteristics were obtained from self‐reports by the pregnant women, and pre‐pregnancy weight and height were retrieved from the medical records to calculate the pre‐pregnancy BMI.^24^

We previously developed a prediction model for PE and superimposed PE (SP) using competing risk model,^30^, ^31^, ^32^ consisting of basic characteristics routinely collected during antenatal care in Japan.^33^ We obtained gestational age at the previous delivery, the inter‐birth interval from the self‐reported data, and blood pressure values from the medical records when integrating nutrition intake into this prediction model. The mean arterial pressure (MAP = [systolic blood pressure + {2 × diastolic blood pressure}]/3) was calculated.^24^

### 2.3 Outcome measures

PE, SP, and gestational hypertension (GH) were identified based on the American College of Obstetricians and Gynecologists (ACOG) guidelines from 2002,^34^ which were the standard guidelines when the TMM BirThree Cohort Study started. We also analyzed the combined outcome of PE and SP (PE+SP) that was used in our previous prediction model.^33^

We defined PE as systolic blood pressure ≥140 mmHg or diastolic blood pressure ≥90 mmHg and proteinuria (≥2+ in the dipstick test) during at least one visit after week 20 of gestation in women with previously normal blood pressure. SP was diagnosed when women with CH developed proteinuria after week 20 of gestation. GH was defined as systolic blood pressure ≥ 140 mmHg or diastolic blood pressure ≥ 90 mmHg, but no proteinuria during at least one visit after week 20 of gestation in women with previously normal blood pressure. These phenotypes were automatically diagnosed by an algorithm based on the medical record data and validated by a physician. Blood pressure measurement and dipstick test for proteinuria were undertaken as per usual protocol in clinics and hospitals and not standardized, as detailed elsewhere.^35^

### 2.4 Statistical analysis

Dietary electrolytes intake with and without energy adjustment was divided into five percentile groups: category 1, <20%; category 2, 20%–39%; category 3, 40%–59%; category 4, 60%–79%; category 5, ≥80%. Category 5 was used as the reference. Basic characteristics and the prevalence of the HDP subtypes were compared among the electrolytes’ quintile groups without energy adjustment using analysis of variance for continuous variables and the chi‐squared or Fisher's exact test for categorical variables.

We performed logistic regression analysis to assess the relationship between the dietary electrolytes intake and the prevalence of the HDP subtypes and PE+SP after adjusting for maternal age (older than 35 years or not), pre‐pregnancy BMI (over 25 kg/m2 or not), smoking status (yes or no), alcohol consumption (yes or no), educational attainment (over 12 years or not), DM, SLE, family history of HDP, family history of CH, parity with or without HDP, and conception by IVF. Pregnant women with CH were included in the population for PE+SP outcome analysis. CH was considered a risk factor for PE+SP and used as a covariate. Pregnant women with CH were excluded from the analysis of PE and GH, while pregnant women without CH were excluded from the analysis of SP. Multiple imputations with five imputed datasets were performed to address missing data in possible confounders using the mice package in R.^36^ Since this cohort was recruited after the Great East Japan Earthquake, we tested the interaction between earthquake damage (damage to houses [total or partial] and death of acquaintances) and electrolyte intake to confirm its generalizability.

We examined the relationship between dietary electrolytes intake and gestational age at delivery with PE+SP to develop the prediction model. We compared models with linear‐, quadratic‐, and broken‐stick‐transformed electrolytes using the Akaike information criterion (AIC) as a measure of goodness of fit. We selected the electrolyte transformation values according to their results and included them in the prediction models.

We performed a parametric survival time analysis that considered deliveries without PE+SP as censored observations, assuming gaussian survival curve distribution. Dietary electrolyte intake and basic maternal characteristics were included in our model. MAP, excluded from the logistic regression model, was included in the prediction model because it could be a strong mediator of PE+SP onset. We used the log_10_‐transformed multiple of the median (log MoM) values of MAP. Educational attainment, smoking status, alcohol consumption, and family history of CH were excluded. Gestational age at the last delivery and the inter‐birth interval in parous women were included in the prediction model based on the findings of a previous study.^30^ Participants with missing data were excluded. All continuous variables were centralized by their mean values before developing the model.

The probability of delivery with PE+SP before specific gestational ages (week 37, 39, and 42 of gestation) was divided into 10 equal‐size probability intervals, and calibration was assessed using a calibration plot with the confidence intervals (CIs) calculated based on 1000 bootstrap re‐samplings. We applied the five‐fold cross‐validation and calculated the area under the receiver operating characteristic curve (AUROC) for each fold. The mean of the five AUROCs (mAUROC) represented the model's performance. We used the bootstrap method to obtain the mAUROC distributions and the difference in mAUROCs among models and assessed 95% CIs. Statistical analysis was performed using R, Version 3.5.3 (https://www.r‐project.org).

## 3 RESULTS

Of the 19 914 included pregnant women, 804 had a history of CH, 547 had PE at parturition, 278 had SP, and 896 had GH. The mean age at gestation week 10–13 antenatal care was 31.7 years, and 9,543 (48%) were nulliparous. The prevalence of PE was 3.5% and 2.6% for participants with a calcium intake of <251 mg/day and > 623 mg/day, and 3.3% and 2.5% for potassium intake of <1313 and >2676 mg/day, respectively (Tables 1 and S1).

**TABLE 1:** Participant characteristics

	<251	251–356	356–465	465–623	>623	*p*‐value
Daily dietary calcium intake, mg	*n* = 3983	*n* = 3983	*n* = 3982	*n* = 3983	*n* = 3983	*n* = 3983
Maternal age, years	30.1 ± 5.2	31.5 ± 5.0	31.9 ± 4.8	32.4 ± 4.8	32.5 ± 4.8	<.001
Body mass index, kg/m2	21.7 ± 3.4	21.5 ± 3.2	21.5 ± 3.0	21.4 ± 3.0	21.5 ± 3.1	<.001
Gestational age, weeks	39.2 ± 1.7	39.2 ± 1.7	39.2 ± 1.6	39.1 ± 1.8	39.1 ± 1.7	.02
Smoking at recruitment, %	158 (4.0)	114 (2.9)	79 (2.0)	71 (1.8)	87 (2.2)	.007
Alcohol use at recruitment, %	680 (17.2)	729 (18.4)	800 (20.1)	838 (21.1)	871 (21.9)	.2
Education for ≤12 years, %	995 (43.2)	889 (34.8)	786 (30.1)	729 (28.2)	734 (28.4)	<.001
Diabetes mellitus (type 1 or 2), %	8 (.3)	7 (.3)	10 (.4)	7 (.3)	15 (.6)	.8
Systemic lupus erythematosus, %	2 (.1)	4 (.2)	4 (.2)	2 (.1)	7 (.3)	.8
Family history of HDP, %	59 (2.5)	72 (2.8)	83 (3.2)	82 (3.1)	79 (3.0)	.6
Family history of CH						
Paternal history of CH, %	514 (22.1)	645 (25.0)	661 (25.1)	675 (25.9)	650 (25.0)	.02
Maternal history of CH, %	387 (16.6)	528 (20.5)	506 (19.2)	513 (19.7)	478 (18.4)	<.001
Brother's history of CH, %	11 (.5)	20 (.8)	27 (1.0)	31 (1.2)	26 (1.0)	.2
Sister's history of CH, %	4 (.2)	12 (.5)	10 (.4)	13 (.5)	10 (.4)	.1
Parity						
Nulliparous, %	2177 (54.7)	2020 (50.8)	1912 (48.1)	1775 (44.7)	1659 (41.7)	.002
Parous with previous HDP, %	84 (2.1)	102 (2.6)	103 (2.6)	99 (2.5)	111 (2.8)	
Parous with no previous HDP, %	1716 (43.1)	1853 (46.6)	1961 (49.3)	2098 (52.8)	2207 (55.5)	
Inter‐birth interval, years	3.7 ± 2.6	3.8 ± 2.6	3.8 ± 2.4	3.7 ± 2.4	3.7 ± 2.3	.3
Last delivery gestational age, weeks	39.0 ± 1.9	38.9 ± 1.8	39.0 ± 1.7	38.9 ± 1.9	38.9 ± 1.9	.3
Conception by IVF, %	130 (3.3)	171 (4.3)	203 (5.1)	216 (5.4)	202 (5.1)	.02
Mean arterial pressure, mmHg	81.1 ± 9.6	80.8 ± 10.0	80.2 ± 9.6	79.9 ± 9.3	79.4 ± 9.7	<.001
Dietary nutritional intake						
Calcium, mg/day	173.4 ± 59.1	304.4 ± 30.4	409.8 ± 31.2	535.9 ± 44.9	1009.5 ± 611.5	<.001
Potassium, mg/day	1079.3 ± 402.6	1606.8 ± 374.0	1947.3 ± 445.3	2325.7 ± 526.5	3445.3 ± 1587.9	<.001
Sodium, mg/day	1887.8 ± 871.6	2681.0 ± 897.3	3120.8 ± 1,030.4	3594.4 ± 1,216.4	4691.2 ± 2553.8	<.001
Magnesium, mg/day	122.3 ± 41.7	172.5 ± 39.5	203.5 ± 47.3	237.5 ± 53.4	333.5 ± 149.1	<.001
Na/K ratio	1.8 ± .6	1.7 ± .4	1.6 ± .4	1.5 ± .4	1.4 ± .4	<.001
Energy, kcal/day	1156.6 ± 384.6	1449.1 ± 366.5	1618.3 ± 376.4	1813.2 ± 442.3	2352.2 ± 94.2	<.001
HDP subtypes						
Not affected	3430 (86.1)	3511 (88.1)	3562 (89.5)	3582 (89.9)	3582 (89.9)	.1
CH	130 (3.3)	113 (2.8)	102 (2.6)	91 (2.3)	90 (2.3)	
Gestational hypertension	209 (5.2)	181 (4.5)	181 (4.5)	166 (4.2)	159 (4.0)	
Pre‐eclampsia	138 (3.5)	116 (2.9)	91 (2.3)	98 (2.5)	104 (2.6)	
Superimposed pre‐eclampsia	76 (1.9)	62 (1.6)	46 (1.2)	46 (1.2)	48 (1.2)	

*Notes*: Data are expressed as means ± standard deviations for continuous variables and *n* (%) for categorical variables. Differences were evaluated by analysis of variance for continuous variables and the chi‐squared or Fisher's exact test for categorical variables.

Abbreviations: CH, chronic hypertension; HDP, hypertensive disorders of pregnancy; IVF, in vitro fertilization; Na/K ratio, sodium‐to‐potassium daily intake ratio. The dietary calcium intakes are shown without energy adjustment.

Logistic regression analysis showed that low crude calcium intake was related to PE prevalence (Table 2; odds ratio (OR), 1.31; 95% CI, 1.00–1.70). The PE trend was significant for crude calcium and energy‐adjusted magnesium intakes (Table 2; *P* for trend, .02 and .03, respectively). SP was not related to any of the assessed nutritional electrolytes; however, PE+SP was related to crude calcium and potassium intake and energy‐adjusted calcium, potassium, and magnesium intake (Table 2; *P* for trend, .01, .048, .02, .04, and .02, respectively). The prevalence of GH was low in participants with low energy‐adjusted Na/K ratio and high in participants with low energy‐adjusted calcium intake (Table 2; OR, .75; 95% CI, .61–.94 and OR, 1.30; 95% CI, 1.04–1.62, respectively). The interactions between the earthquake damage and electrolytes intake were not significant, except for energy‐adjusted magnesium and house damage (*P* for interaction, .007). Furthermore, stratified by house damage, OR of energy‐adjusted magnesium intake (quintile 1 [<183 mg/day] versus quintile 5 [>243 mg/day]) on PE+SP was 1.45 (1.03–2.05) in the participants without house damage and 1.09 (.78–1.53) in the participants with house damage.

**TABLE 2:** The relationship between dietary electrolytes intake and the hypertensive disorders of pregnancy subtypes

	PE			SP			PE+SP			Gestational hypertension		
Quintiles	OR (95% CI)	*p*‐value	*P* for trend	OR (95% CI)	*p*‐value	*P* for trend	OR (95% CI)	*p*‐value	*P* for trend	OR (95% CI)	*p*‐value	*P* for trend
Crude												
Calcium intake in mg/day												
<251	1.31 (1.00–1.70)	.049	.02	1.08 (.67–1.73)	.8	.6.	1.28 (1.02–1.62)	.04	.01	1.25 (1.01–1.56)	.04	.04
251–356	1.07 (.81–1.41)	.6		1.11 (.68–1.81)	.7		1.09 (.86–1.39)	.5		1.10 (.88–1.37)	.4	
356–465	.85 (.64–1.14)	.3		.89 (.53–1.48)	.7		.90 (.70–1.15)	.4		1.12 (.90–1.40)	.3	
465–623	.93 (.70–1.23)	.6		.99 (.59–1.65)	.96		.95 (.75–1.22)	.7		1.04 (.83–1.30)	.7	
>623	Reference	–		Reference	–		Reference	–		Reference	–	
Potassium intake in mg/day												
<1313	1.30 (.99–1.70)	.06	.06	1.05 (.65–1.71)	.8	.9	1.26 (1.00–1.60)	.05	.048	1.12 (.90–1.38)	.3	.2
1313–1704	1.11 (.84–1.47)	.5		1.06 (.65–1.73)	.8		1.15 (.90–1.46)	.3		1.08 (.87–1.34)	.5	
1704–2104	1.01 (.76–1.35)	.9		.82 (.49–1.38)	.5		.99 (.77–1.27)	.9		.95 (.76–1.19)	.7	
2104–2676	1.05 (.79–1.39)	.7		1.11 (.66–1.86)	.7		1.09 (.85–1.39)	.5		.98 (.79–1.23)	.9	
>2676	Reference	–		Reference	–		Reference	–		Reference	–	
Sodium intake in mg/day												
<1992	1.15 (.87–1.51)	.3	.4	.96 (.59–1.56)	.9	.96	1.11 (.88–1.41)	.4	.4	1.02 (.83–1.26)	.8	.8
1992–2621	1.03 (.78–1.36)	.8		1.14 (.69–1.88)	.6		1.09 (.86–1.39)	.5		.92 (.74–1.14)	.4	
2621–3263	1.05 (.80–1.39)	.7		1.19 (.72–1.99)	.5		1.08 (.85–1.38)	.5		.98 (.80–1.22)	.9	
3263–4170	1.03 (.78–1.36)	.8		1.01 (.60–1.70)	.98		1.05 (.82–1.35)	.7		.90 (.73–1.12)	.4	
>4170	Reference	–		Reference	–		Reference	–		Reference	–	
Magnesium intake in mg/day												
<142	1.28 (.98–1.68)	.07	.1	1.12 (.69–1.80)	.6	.9	1.25 (.99–1.58)	.06	.1	1.11 (.90–1.38)	.3	.2
142–179	1.06 (.80–1.40)	.7		1.06 (.64–1.75)	.8		1.09 (.86–1.39)	.5		1.05 (.85–1.31)	.6	
179–217	.95 (.72–1.27)	.7		.85 (.52–1.40)	.5		.94 (.74–1.21)	.6		.99 (.80–1.23)	.9	
217–272	1.00 (.75–1.32)	.98		1.27 (.76–2.14)	.4		1.07 (.84–1.36)	.6		.95 (.76–1.19)	.7	
>272	Reference	–		Reference	–		Reference	–		Reference	–	
Na/K ratio												
<1.23	.86 (.65–1.14)	.3	.3	1.13 (.69–1.84)	.6	.6	.91 (.72–1.16)	.5	.5	.78 (.63–.97)	.03	.01
1.23–1.45	1.12 (.86–1.47)	.4		1.27 (.77–2.09)	.3		1.14 (.90–1.44)	.3		.90 (.72–1.11)	.3	
1.45–1.65	.89 (.67–1.17)	.4		1.39 (.84–2.30)	.2		1.00 (.78–1.27)	.99	.94 (.77–1.17)	.6	
1.65–1.91	1.15 (.88–1.50)	.3		1.15 (.70–1.88)	.6		1.13 (.89–1.43)	.3		1.05 (.86–1.30)	.6	
>1.91	Reference	–		Reference	–		Reference	–		Reference	–	
Energy in Kcal/day												
<1,215	.94 (.72–1.23)	.6	.9	1.00 (.64–1.56)	.98	.98	.97 (.77–1.22)	.8	.96	1.12 (.91–1.37)	.3	.9
1215–1461	.97 (.74–1.26)	.8		1.03 (.65–1.64)	.9		1.01 (.80–1.27)	.9		.84 (.67–1.05)	.1	
1,461–1,708	.82 (.62–1.08)	.2		.76 (.45–1.27)	.3		.82 (.64–1.05)	.1		.86 (.69–1.07)	.2	
1708–2053	.87 (.66–1.14)	.3		1.03 (.63–1.68)	.9		.94 (.74–1.19)	.6		1.05 (.85–1.30)	.6	
>2053	Reference	–		Reference	–		Reference	–		Reference	–	
Energy‐adjusted												
Calcium intake in mg/day												
<330	1.17 (.88 to 1.56)	.3	.06	1.17 (.73 to 1.87)	.5	.4	1.18 (.92–1.51)	.2	.02	1.30 (1.04 to 1.62)	.02	.01
330–420	1.48 (1.13–1.94)	.01		1.32 (.82–2.13)	.3		1.45 (1.15–1.84)	.002		1.31 (1.05–1.63)	.02	
420–496	1.09 (.82–1.46)	.6		1.12 (.68–1.85)	.7		1.09 (.85–1.39)	.5		1.11 (.89–1.39)	.4	
496–599	1.13 (.85–1.51)	.4		1.13 (.68–1.88)	.6		1.14 (.89–1.46)	.3		1.13 (.90–1.41)	.3	
>599	Reference	–		Reference	–		Reference	–		Reference	–	
Potassium intake in mg/day												
<1714	1.27 (.97–1.66)	.08	.06	1.06 (.66–1.69)	.8	.7	1.23 (.97–1.55)	.09	.04	1.43 (1.14–1.79)	0 .002	.004
1714–1949	.99 (.74–1.31)	.9		1.23 (.75–2.02)	.4		1.07 (.84–1.36)	.6		1.23 (.97–1.54)	.08	
1949–2150	1.17 (.89–1.54)	.3		1.02 (.62–1.69)	.9		1.15 (.90–1.46)	.3		1.50 (1.20–1.88)	<.001	
2150–2423	.89 (.67–1.19)	.4		1.04 (.63–1.73)	.9		.94 (.73–1.20)	.6		1.18 (.93–1.49)	.2	
>2423	Reference	–		Reference	–		Reference	–		Reference	–	
Sodium intake in mg/day												
<2504	1.15 (.87–1.52)	.3	.1	1.24 (.76–2.02)	.4	.6	1.19 (.94–1.52)	.2	.06	.94 (.75–1.17)	.6	.1
2504–2946	1.26 (.96–1.65)	.09		1.32 (.79–2.18)	.3		1.30 (1.03–1.66)	.03		.91 (.73–1.13)	.4	
2,946–3,332	1.01 (.76–1.34)	.9		1.37 (.82–2.30)	.2		1.09 (.85–1.40)	.5		1.03 (.83–1.28)	.8	
3332–3835	1.02 (.76–1.35)	.9		1.35 (.81–2.24)	.2		1.11 (.86–1.42)	.4		1.16 (.94–1.43)	.2	
>3835	Reference	–		Reference	–		Reference	–		Reference	–	
Magnesium in mg/day												
<183	1.25 (.95–1.64)	.1	.03	1.18 (.73–1.91)	.5	.5	1.26 (.99–1.59)	.06	.02	1.24 (1.00–1.55)	.05	.04
183–203	1.20 (.91–1.59)	.2		1.34 (.83–2.17)	.2		1.26 (.99–1.60)	.06		1.23 (.99–1.53)	.07	
203–220	1.09 (.82–1.44)	.6		1.08 (.65–1.78)	.8		1.09 (.86–1.40)	.5		1.17 (.93–1.46)	.2	
220–243	.95 (.71–1.27)	.7		1.26 (.76–2.10)	.4		1.04 (.81–1.33)	.8		1.12 (.90–1.41)	.3	
>243	Reference	–		Reference	–		Reference	–		Reference	–	
Na/K ratio												
<1.23	.91 (.69–1.20)	.5	.4	1.05 (.64–1.72)	.8	.7	.93 (.73–1.19)	.6	.6	.75 (.61–.94)	.01	.005
1.23–1.45	1.11 (.85–1.46)	.4		1.40 (.85 to 2.31)	.2		1.16 (.92–1.47)	.2		.82 (.66–1.02)	.07	
1.45–1.65	.86 (.64–1.14)	.3		1.41 (.85 to 2.33)	.2		.98 (.77–1.24)	.8		.93 (.75–1.14)	.5	
1.65–1.91	1.18 (.91–1.54)	.2		1.14 (.70 to 1.87)	.6		1.16 (.91–1.46)	.2		.95 (.78–1.17)	.6	
>1.91	Reference	–		Reference	–		Reference	–		Reference	–	

*Notes*: The independent variables (the electrolytes intake) were adjusted for maternal age, pre‐pregnancy body mass index, smoking status, alcohol consumption status, educational attainment, diabetes mellitus, systemic lupus erythematosus, family history of hypertensive disorders of pregnancy, family history of chronic hypertension, parity with or without hypertensive disorders of pregnancy, and conception by in vitro fertilization.

Pregnant women with chronic hypertension (CH) were included in the analysis with PE+SP as the outcome in the population, considered a risk factor for SP, and used as a covariate. Pregnant women with CH were excluded from the analysis with PE or gestational hypertension as the outcome, while pregnant women without CH were excluded from the analysis with SP as the outcome.

Abbreviations: CI: confidence interval; Na/K ratio, sodium‐to‐potassium intake ratio; OR, odds ratio; PE, pre‐eclampsia; PE+SP, pre‐eclampsia and superimposed pre‐eclampsia; SP, superimposed pre‐eclampsia.

After excluding participants with missing data, the prediction models were based on data of 9208 pregnant women. We fitted dietary electrolytes intake to the gestational age at delivery with PE+SP (Figure S1). Based on the resulting plots and goodness of fit in the AIC (Table S2), we selected the broken‐stick model for crude calcium, potassium, sodium, and magnesium, and energy‐adjusted calcium; we chose the quadratic model for crude Na/K ratio and energy, and energy‐adjusted potassium, sodium, magnesium, and Na/K ratio.

**FIGURE 1:**
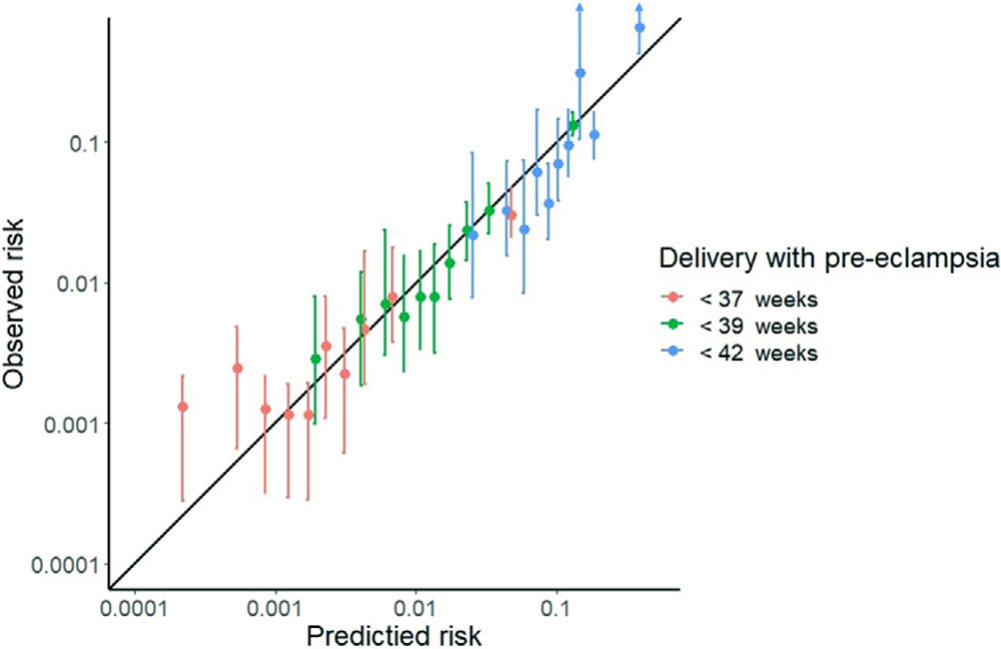
Pre‐eclampsia prediction calibration plot. The calibration plot reveals the relationship between the predicted and observed rates of deliveries with pre‐eclampsia or superimposed pre‐eclampsia before gestational week 37 (red), 39 (blue), and 42 (green) divided into ten equal‐sized intervals. The diagonal line indicates the performance of an ideal model. The vertical lines in all data points represent the 95% confidence intervals estimated using 1000 bootstrap resampling

The mean gestational age at delivery of participants with PE+SP when using a model that included crude calcium intake in the reference population (35 years old, BMI 21.4 kg/m2, no medical history, no family history of HDP, nulliparous, spontaneous conception, MAP 80 mmHg, 400 mg of daily dietary calcium intake) was 46.5 (95% CI, 45.8–47.1) weeks (Table 3). We confirmed that the model was satisfactorily calibrated (Figure 1), particularly when predicting for participants with gestational ages <39 and <42 weeks at parturition. The bootstrap simulation found mAUROC of .779 (95% CI, .745–.800) and .779 (95% CI, .745–.800) for models with crude calcium and potassium intake, respectively, which were significantly higher than the model with only the basic characteristics and MAP (Table 4). A prediction model with calcium and potassium together resulted in mAUROC of .776 (95% CI, .738–.794).

**TABLE 3:** A prediction model for delivery with pre‐eclampsia or superimposed pre‐eclampsia that included dietary calcium intake as a predictor variable

	Coefficients	95% confidence interval
Maternal age, years	–.1	–.2 to –.01
Body mass index, kg/m2	–.06	–.1 to –.01
Chronic hypertension	–2.4	–3.0 to –1.8
Diabetes mellitus (types 1 and 2)	–1.2	–3.0 to .5
Systemic lupus erythematosus	–5.4	–8.1 to –2.7
Family history of HDP	–1.2	–2.0 to –.4
Parity		
Parous with previous HDP	–3.2	–4.1 to –2.4
Parous with no previous HDP	–.7	–1.3 to –.1
Inter‐birth interval, years	–.1	–.2 to –.01
Last delivery gestational age, weeks	.5	.3 to .8
Conception by in vitro fertilization	–.02	–.7 to .6
log MoM of the MAP	–16.9	–20.8 to –12.9
Calcium intake, 100 mg/day	–.2	–.3 to –.06
Constant	46.5	45.8 to 47.1

*Notes*: The broken‐stick model was used for maternal age, gestational age of last delivery in weeks, and dietary calcium intake. Maternal age minus 35 was included as a covariate if the maternal age was >35. Gestational age of last delivery minus 37 was included if delivery was before week 37. Dietary calcium intake minus 600 was included if daily consumption was <600 mg. Body mass index and interval were centralized to their means (21.4 and 1.8 kg/m2, respectively).

Abbreviations: HDP, hypertensive disorders of pregnancy; log MoM, log_10_ transformed multiple of the median; MAP, mean arterial pressure.

**TABLE 4:** The prediction models’ performance

		mAUROC (95% CI)	Inter‐model mAUROC (95% CI) difference
Without the electrolyte values (reference)		.774 (.738–.794)	
	Calcium	.779 (.745–.800)	.006 (.0003 to .01)
	Potassium	.779 (.745–.800)	.005 (.0004 to .01)
With crude electrolyte values			
	Sodium	.777 (.743–.797)	.004 (–.002 to .01)
	Magnesium	.777 (.743–.799)	.004 (–.0007 to .01)
	Na/K ratio	.771 (.735–.790)	–.002 (–.007 to –.0002)
	Energy	.774 (.739–.794)	.001 (–.004 to .005)
With energy‐adjusted electrolyte values	Calcium	.774 (.739–.794)	.00005 (–.002 to .003)
	Potassium	.773 (.737–.792)	–.001 (–.005 to .002)
	Sodium	.772 (.736–.791)	–.001 (–.007 to .002)
	Magnesium	.773 (.738–.793)	–.0007 (–.003 to .002)
	Na/K ratio	.771 (.735–.790)	–.002 (–.007 to –.0001)

*Note*: Differences between models with and without the electrolyte values are shown in the rightmost column.

Abbreviations: CI, confidence interval; mAUROC, mean area under the receiver operating characteristic curve.

## 4 DISCUSSION

This study found that lower crude calcium intake was related to the prevalence of PE, and the trend was significant for crude calcium and energy‐adjusted magnesium intake. SP was not related to any nutritional electrolyte intake; however, PE+SP was related to crude calcium and potassium and energy‐adjusted calcium, potassium, and magnesium intake. A prediction model for PE+SP that included dietary calcium or potassium intake performed better than a model based only on the basic characteristics and MAP.

The World Health Organization^37^ recommended calcium supplementation based on a large meta‐analysis,^9^ especially in populations with low calcium intake, leading to the assumption that adequate dietary calcium intake should reduce the risk of PE.^10^, ^11^ Our results showed that pregnant women with low calcium intake were at a higher risk of PE, consistent with the results of a calcium supplementation trial^9^ and some observational studies.^10^ Adding data on calcium intake to the prediction model slightly improved its performance. No previous study had developed a prediction model that included dietary calcium intake. Our results suggested the usefulness of performing FFQ early in the pregnancy to identify women at high risk of PE. Potassium intake showed the same tendency as calcium in logistic regression analysis and the prediction of PE+SP. Previous studies suggested that potassium intake reduced systolic and diastolic blood pressure in CH^38^; however, its relationship with PE remained unclear.^12^ Our study was the first to suggest a role for potassium intake in the pathogenesis of PE. Prediction models that included calcium or potassium intake performed similarly, and a prediction model with both was not better than either. We assumed this finding was partly because of overfitting and partly because the predictive value of potassium intake overlapped with that of calcium. Potassium and calcium affect blood pressure through several mechanisms, in some of which they work together.^39^ Additionally, low calcium and potassium intakes might reflect the same dietary pattern or daily habits. Therefore, these electrolytes share information that could help predict PE+SP. We would also like to emphasize that crude, but not energy‐adjusted, calcium and potassium could improve the prediction model, suggesting that absolute intake was a more important determinant for PE+SP than the intake relative to the energy.

Sodium intake was not associated with any of the HDP subtypes. While it is a significant risk factor for CH,^40^ its relationship with PE onset remains unclear.^41^ Previous studies reported higher sodium intake in pregnant women with PE than those without PE,^12^ but a meta‐analysis demonstrated that reduced dietary sodium intake did not decrease the onset of PE.^13^ The Na/K ratio was not associated with the onset of PE, possibly because sodium retention does not play a major role in the pathogenesis of PE.^42^ However, the Na/K ratio was related to GH, possibly indicating that GH pathogenesis was similar to that of CH.^26^ For magnesium, the trend in the odds ratios was linear in PE, PE/SP, and GH, and significant. However, no significant risk was noted in any quintile group, suggesting that this study may have been underpowered. The larger effect size for PE+SP when restricted to participants without house damage by the earthquake suggests that the impact may be larger in the general population. A meta‐analysis of small studies did not find a significant preventive effect of magnesium supplementation on PE.^14^ Further interventional studies are needed, especially in the low magnesium intake population.

The similarity between PE and PE+SP results and the lack of similarity between these and SP results may be due to the difference in sample size, with PE participants being twice as many as SP participants. When the interaction between CH and electrolyte intake was analyzed for PE+SP under the same conditions in Table 2, no significant interaction was found (P for interaction, calcium: .90, potassium: .83); therefore, the effect of heterogeneity between PE and SP might be limited.

### 4.1 Clinical implications

Our results have at least two implications. First, they shed light on the contentious relationship between dietary calcium intake and PE. A calcium‐rich diet and calcium supplementation, both less invasive interventions than medication, might prevent PE onset. Additionally, our results hint at the possibility that dietary potassium intake affects PE onset, suggesting the need for comprehensive nutrition counseling that incorporates both the potassium and calcium results. Second, our prediction model included only variables measured at regular antenatal visits and data on dietary electrolytes intake calculated based on data from FFQs. The FFQ can be conducted without additional facilities or human resources. Therefore, our model can be easily implemented in clinical practice. Previous studies^43^ reported larger AUROCs using models with uncommon biomarkers in Japan such as uterine artery blood flow assessed by Doppler ultrasonography.^31^ However, given that these are uncommon in clinical settings, we considered our model more practical than these previous models. We recommend that all women complete an FFQ early in their pregnancy to identify those at a high risk of developing PE so they can undergo intensive follow‐up with nutrition counseling throughout their gestation.

### 4.2 Research implications

Of the information available on nutritional intake from the FFQ, this study focused only on electrolytes, highlighting the FFQ's effectiveness. We hope further studies will help establish a better prediction model with other nutritional intake data retrieved from the FFQ, including over a hundred food items and tens of nutrition choices. Besides, only a few nutritional intervention studies for PE have been performed.^13^, ^44^ Therefore, our study could motivate researchers to conduct nutritional trials examining factors such as potassium‐rich diets and nutrition counseling, which were shown effective for hypertension control in hypertensive non‐pregnant adults.^45^, ^46^ Besides, our results highlight the value of nutritional intervention during the preconception period.

### 4.3 Strengths and limitations

The strength of the present study is that it was the first to describe the relationship between dietary calcium intake and the HDP subtypes and develop a prediction model for PE+SP that included electrolyte intake data from an FFQ. Our cohort was relatively large and included almost half of the newborns in the Miyagi Prefecture (8605 of 17 347 newborns) in 2016.^21^ However, the present study had some limitations. First, the definition of PE differed from the commonly accepted definition^47^ in that blood pressure was measured only once and the diagnosis was made automatically using a computer algorithm. In addition, the procedures used for the blood pressure and proteinuria measurements were not standardized among hospitals and clinics. However, the prevalence of PE in the present study was similar to that of previous studies (2.7% in our study, 2.7% in a previous Japanese study,^48^ 2.9% in Sweden,^49^ 2.3% in China,^49^ and 3.4% in USA^3^), suggesting that our diagnoses were valid. Second, while pregnant women may change their dietary pattern during gestation, we assumed it was constant during the study period and did not distinguish between various timings of taking the FFQ. A previous study investigating the validity of the FFQ during early gestation in Japan showed good consistency with a 3‐day dietary recording.^50^ However, no study has investigated the validity of the FFQ throughout gestation. Our study population consumed almost the same amount of calcium (486.6 vs. 493.7 mg/day) as in the reported 3‐day dietary recording,^50^ suggesting that our calcium intake assessment method and results related to calcium intake were valid. However, potassium intake in our study was lower than that previously reported (2080.9 vs. 2378.8 mg).^50^ Therefore, our results might be biased concerning potassium intake, or they might reflect differences in study population characteristics. Further, 24‐hour urine testing, the gold standard for assessing sodium and potassium intake, was not performed in the present study. Third, since the SP population had pre‐existing hypertension or kidney disease in early pregnancy, it is possible that they were already receiving therapeutic interventions, including dietary modifications, and causal reversals need to be considered. However, no major directional changes were observed with respect to our estimates. Fourth, we did not account for electrolytes intake through medication or supplements. Therefore, we may have underestimated the actual electrolytes intake.

## 5 CONCLUSIONS

Low dietary calcium, potassium, and magnesium were associated with a higher prevalence of various HDP subtypes. The developed prediction model implied that absolute calcium or potassium intake might play a key role in PE+SP pathogenesis.

## AUTHOR CONTRIBUTIONS

Hisashi Ohseto wrote the original draft. Hisashi Ohseto, Mami Ishikuro, and Taku Obara carried out the statistical analysis. Mami Ishikuro, Taku Obara, Keiko Murakami, Tomomi Onuma, Aoi Noda, Ippei Takahashi, Fumiko Matsuzaki, Fumihiko Ueno, Noriyuki Iwama, Masahiro Kikuya, Hirohito Metoki, Junichi Sugawara, and Shinichi Kuriyama were involved in the acquisition, interpretation of data, and the manuscript review process. All authors approved the submitted version.

## CONFLICT OF INTEREST

None.

## Supporting information

Supporting Information

Supporting Information

Supporting Information
