# Supplementary figures and images for: Dietary calcium intake was related to the onset of pre‐eclampsia: The TMM BirThree Cohort Study
Source: J Clin Hypertens (Greenwich). 2022 Dec 28;25(1):61–70. doi: 10.1111/jch.14606 (PMC9832228; doi:10.1111/jch.14606)

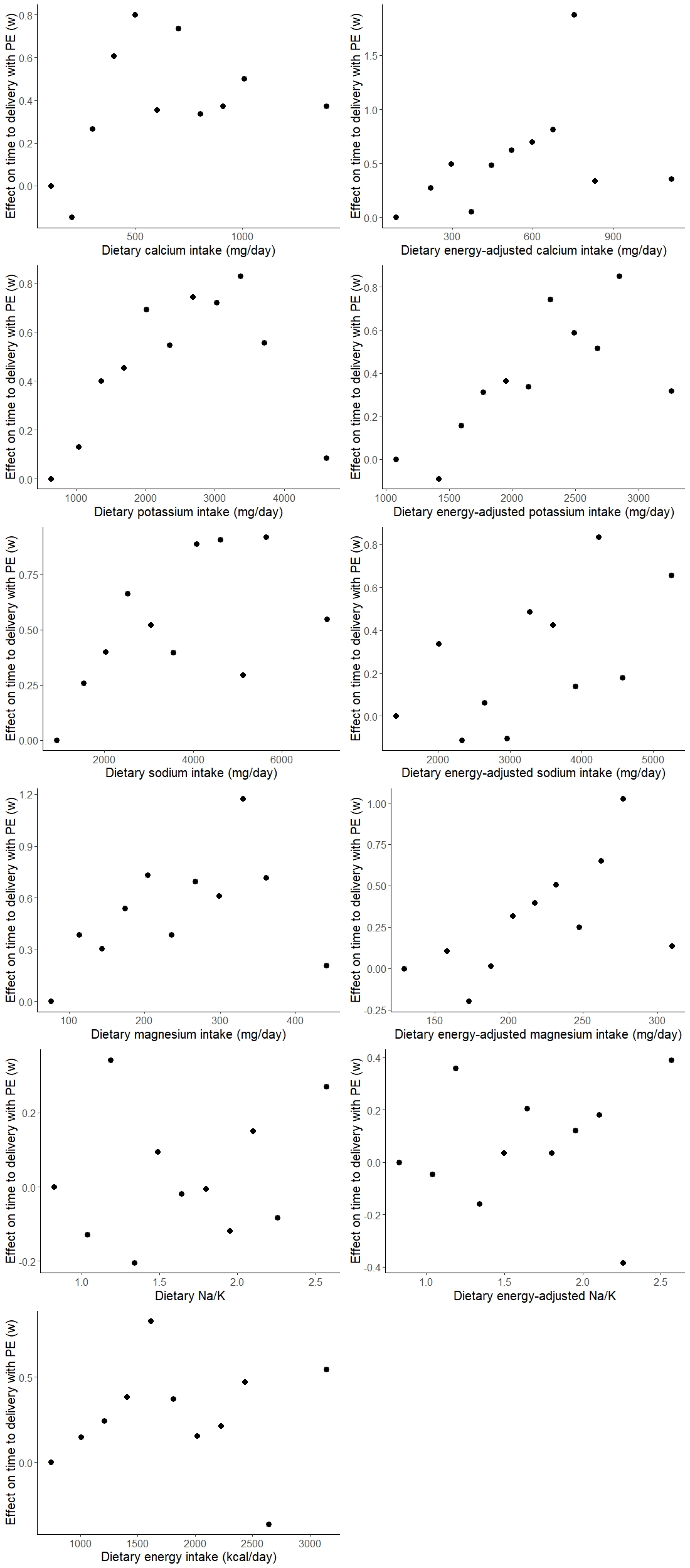

Supplement: Supplementary file 1 — Supporting Information [file JCH-25-61-s001.jpeg]
