# Supplementary material for: Dietary calcium intake was related to the onset of pre‐eclampsia: The TMM BirThree Cohort Study
Source: J Clin Hypertens (Greenwich). 2022 Dec 28;25(1):61–70. doi: 10.1111/jch.14606 (PMC9832228; doi:10.1111/jch.14606)
Supplement: Supplementary file 2 — Supporting Information [file JCH-25-61-s002.docx]

Supplemental Table 1. Participant characteristics

| Daily dietary potassium intake, mg | | | <1,313 | 1,313–1,704 | 1,704–2,104 | 2,104–2,676 | >2,676 | *P*-value |
| --- | --- | --- | --- | --- | --- | --- | --- | --- |
|  |  |  | *n*=3,983 | *n*=3,983 | *n*=3,982 | *n*=3,983 | *n*=3,983 |  |
|  | Maternal age, years | | 30.2±5.2 | 31.5±4.9 | 32.0±4.8 | 32.3±4.8 | 32.6±4.8 | <0.0001 |
|  | Body mass index, kg/m^2^ | | 21.6±3.3 | 21.5±3.2 | 21.4±3.0 | 21.5±3.1 | 21.6±3.1 | 0.6 |
|  | Gestational age, weeks | | 39.2±1.7 | 39.2±1.7 | 39.1±1.7 | 39.1±1.8 | 39.1±1.6 | 0.005 |
|  | Current smoking at recruitment, % | | 139 (3.5) | 93 (2.3) | 89 (2.2) | 95 (2.4) | 93 (2.3) | 0.003 |
|  | Current alcohol use at recruitment, % | | 655 (16.6) | 715 (18.0) | 805 (20.3) | 849 (21.4) | 894 (22.5) | 0.09 |
|  | Education ≤ 12 years, % | | 964 (40.8) | 840 (33.2) | 817 (31.1) | 740 (28.5) | 772 (30.7) | <0.0001 |
|  | Diabetes mellitus (type 1 or 2), % | | 8 (0.3) | 10 (0.4) | 5 (0.2) | 11 (0.4) | 13 (0.5) | 0.9 |
|  | Systemic lupus erythematosus, % | | 1 (0.0) | 5 (0.2) | 3 (0.1) | 6 (0.2) | 4 (0.2) | 0.3 |
|  | Family history of HDP, % | | 55 (2.3) | 76 (3.0) | 75 (2.8) | 86 (3.3) | 83 (3.3) | 0.2 |
|  | Family history of CH | |  |  |  |  |  |  |
|  |  | Paternal history of CH, % | 519 (21.7) | 602 (23.6) | 677 (25.6) | 730 (27.8) | 617 (24.3) | 0.1 |
|  |  | Maternal history of CH, % | 385 (16.1) | 483 (18.9) | 533 (20.2) | 530 (20.2) | 481 (19.0) | 0.01 |
|  |  | Brother's history of CH, % | 13 (0.5) | 21 (0.8) | 24 (0.9) | 28 (1.1) | 29 (1.1) | 0.3 |
|  |  | Sister's history of CH, % | 5 (0.2) | 12 (0.5) | 6 (0.2) | 15 (0.6) | 11 (0.4) | 0.2 |
|  | Parity | |  |  |  |  |  |  |
|  |  | Nulliparous, % | 2,306 (58.1) | 2,031 (51.1) | 1,857 (46.7) | 1,726 (43.4) | 1,623 (40.8) | <0.0001 |
|  |  | Parous with previous HDP, % | 80 (2.0) | 109 (2.7) | 90 (2.3) | 114 (2.9) | 106 (2.7) |  |
|  |  | Parous with no previous HDP, % | 1,586 (39.9) | 1,837 (46.2) | 2,030 (51.0) | 2,134 (53.7) | 2,248 (56.5) |  |
|  |  | Inter-birth interval, years | 3.8±2.6 | 3.7±2.5 | 3.7±2.4 | 3.8±2.5 | 3.7±2.4 | 0.9 |
|  |  | Last delivery gestational age, weeks | 38.9±1.8 | 38.9±1.8 | 39.0±1.8 | 38.9±2.0 | 38.9±1.8 | 0.3 |
|  | Conception by *in vitro* fertilization, % | | 154 (3.9) | 188 (4.7) | 186 (4.7) | 174 (4.4) | 220 (5.5) | 0.07 |
|  | Mean arterial pressure, mmHg | | 81.1±9.7 | 80.9±9.6 | 79.9±9.8 | 80.0±9.6 | 79.5±9.6 | <0.0001 |
|  | Dietary nutrition intake | |  |  |  |  |  |  |
|  |  | Calcium, mg/day | 201.0±92.6 | 330.9±100.5 | 425.0±123.6 | 537.1±168.8 | 939.0±644.3 | <0.0001 |
|  |  | Potassium, mg/day | 971.8±286.3 | 1,511.9±112.2 | 1,895.2±114.9 | 2,360.9±163.0 | 3,664.5±1,468.8 | <0.0001 |
|  |  | Sodium, mg/day | 1,691.8±689.4 | 2,475.2±672.6 | 3,016.3±738.3 | 3,647.7±927.1 | 5,144.2±2,420.3 | <0.0001 |
|  |  | Magnesium, mg/day | 111.4±31.1 | 163.1±19.8 | 198.4±22.2 | 241.5±27.8 | 355.0±138.1 | <0.0001 |
|  |  | Na/K ratio | 1.7±0.6 | 1.6±0.4 | 1.6±0.4 | 1.5±0.4 | 1.4±0.4 | <0.0001 |
|  |  | Energy, kcal/day | 1,069.8±301.3 | 1,397.2±259.9 | 1,596.6±279.7 | 1,846.7±346.1 | 2,479.2±906.6 | <0.0001 |
|  | HDP subtypes | |  |  |  |  |  |  |
|  |  | Not affected | 3,461 (86.9) | 3,506 (88.0) | 3,560 (89.4) | 3,564 (89.5) | 3,576 (89.8) | 0.6 |
|  |  | CH | 119 (3.0) | 113 (2.8) | 113 (2.8) | 92 (2.3) | 89 (2.2) |  |
|  |  | Gestational hypertension | 202 (5.1) | 190 (4.8) | 163 (4.1) | 169 (4.2) | 172 (4.3) |  |
|  |  | Pre-eclampsia | 131 (3.3) | 114 (2.9) | 99 (2.5) | 104 (2.6) | 99 (2.5) |  |
|  |  | Superimposed pre-eclampsia | 70 (1.8) | 60 (1.5) | 47 (1.2) | 54 (1.4) | 47 (1.2) |  |
|  |  |  |  |  |  |  |  |  |
| Daily dietary sodium intake, mg | | | <1,992 | 1,992–2,621 | 2,621–3,263 | 3,263–4,170 | >4,170 | *P*-value |
|  |  |  | *n*=3,983 | *n*=3,983 | *n*=3,982 | *n*=3,983 | *n*=3,983 |  |
|  | Maternal age, years | | 30.6±5.2 | 31.7±4.9 | 32.0±4.9 | 32.1±4.9 | 32.2±4.9 | <0.0001 |
|  | Body mass index, kg/m^2^ | | 21.7±3.3 | 21.5±3.1 | 21.4±3.0 | 21.5±3.1 | 21.6±3.2 | 0.7 |
|  | Gestational age, weeks | | 39.2±1.6 | 39.2±1.8 | 39.2±1.8 | 39.1±1.7 | 39.1±1.6 | 0.0009 |
|  | Current smoking at recruitment, % | | 138 (3.5) | 88 (2.2) | 91 (2.3) | 87 (2.2) | 105 (2.6) | 0.0009 |
|  | Current alcohol use at recruitment, % | | 693 (17.5) | 755 (19.0) | 814 (20.5) | 803 (20.2) | 853 (21.5) | 0.09 |
|  | Education ≤ 12 years, % | | 862 (36.2) | 824 (32.0) | 801 (30.9) | 820 (31.5) | 826 (33.4) | 0.002 |
|  | Diabetes mellitus (type 1 or 2), % | | 9 (0.4) | 10 (0.4) | 11 (0.4) | 8 (0.3) | 9 (0.4) | >0.99 |
|  | Systemic lupus erythematosus, % | | 2 (0.1) | 4 (0.2) | 5 (0.2) | 7 (0.3) | 1 (0.0) | 0.8 |
|  | Family history of HDP, % | | 60 (2.5) | 69 (2.7) | 77 (2.9) | 78 (3.0) | 91 (3.6) | 0.8 |
|  | Family history of CH | |  |  |  |  |  |  |
|  |  | Paternal history of CH, % | 557 (23.1) | 628 (24.1) | 667 (25.5) | 668 (25.4) | 625 (25.0) | 0.4 |
|  |  | Maternal history of CH, % | 425 (17.6) | 476 (18.3) | 540 (20.6) | 487 (18.5) | 484 (19.4) | 0.6 |
|  |  | Brother's history of CH, % | 18 (0.7) | 26 (1.0) | 19 (0.7) | 26 (1.0) | 26 (1.0) | 0.4 |
|  |  | Sister's history of CH, % | 8 (0.3) | 8 (0.3) | 8 (0.3) | 12 (0.5) | 13 (0.5) | >0.99 |
|  | Parity | |  |  |  |  |  |  |
|  |  | Nulliparous, % | 2,313 (58.2) | 2,022 (50.9) | 1,922 (48.3) | 1,723 (43.3) | 1,563 (39.3) | <0.0001 |
|  |  | Parous with previous HDP, % | 96 (2.4) | 105 (2.6) | 96 (2.4) | 97 (2.4) | 105 (2.6) |  |
|  |  | Parous with no previous HDP, % | 1,565 (39.4) | 1,846 (46.5) | 1,960 (49.3) | 2,156 (54.2) | 2,308 (58.0) |  |
|  |  | Inter-birth interval, years | 3.8±2.6 | 3.7±2.6 | 3.8±2.5 | 3.7±2.4 | 3.7±2.4 | 0.3 |
|  |  | Last delivery gestational age, weeks | 38.9±1.8 | 38.9±1.8 | 38.9±1.9 | 38.9±1.9 | 38.9±1.8 | >0.99 |
|  | Conception by *in vitro* fertilization, % | | 167 (4.2) | 192 (4.8) | 207 (5.2) | 173 (4.3) | 183 (4.6) | 0.2 |
|  | Mean arterial pressure, mmHg | | 81.0±10.0 | 80.8±9.7 | 80.1±9.5 | 79.8±9.6 | 79.7±9.6 | <0.0001 |
|  | Dietary nutrition intake | |  |  |  |  |  |  |
|  |  | Calcium, mg/day | 257.7±178.7 | 377.5±205.9 | 458.2±260.3 | 552.0±324.3 | 787.4±622.4 | <0.0001 |
|  |  | Potassium, mg/day | 1,127.5±466.1 | 1,622.6±452.3 | 1,944.1±511.6 | 2,355.0±637.8 | 3,355.1±1,593.7 | <0.0001 |
|  |  | Sodium, mg/day | 1,455.6±446.5 | 2,312.5±179.7 | 2,932.5±184.6 | 3,671.3±258.5 | 5,603.3±2,184.1 | <0.0001 |
|  |  | Magnesium, mg/day | 124.1±46.3 | 171.2±44.3 | 201.7±46.1 | 240.4±59.7 | 331.9±145.8 | <0.0001 |
|  |  | Na/K ratio | 1.4±0.6 | 1.5±0.4 | 1.6±0.4 | 1.7±0.4 | 1.8±0.5 | <0.0001 |
|  |  | Energy, kcal/day | 1,118.3±349.2 | 1,418.8±323.2 | 1,602.0±331.6 | 1,835.7±393.6 | 2,414.6±926.9 | <0.0001 |
|  | HDP subtypes | |  |  |  |  |  |  |
|  |  | Not affected | 3,456 (86.8) | 3,534 (88.7) | 3,535 (88.8) | 3,575 (89.8) | 3,567 (89.6) | 0.09 |
|  |  | CH | 140 (3.5) | 107 (2.7) | 102 (2.6) | 91 (2.3) | 86 (2.2) |  |
|  |  | Gestational hypertension | 196 (4.9) | 172 (4.3) | 180 (4.5) | 164 (4.1) | 184 (4.6) |  |
|  |  | Pre-eclampsia | 122 (3.1) | 109 (2.7) | 109 (2.7) | 105 (2.6) | 102 (2.6) |  |
|  |  | Superimposed pre-eclampsia | 69 (1.7) | 61 (1.5) | 56 (1.4) | 48 (1.2) | 44 (1.1) |  |
|  |  |  |  |  |  |  |  |  |
| Daily dietary magnesium intake, mg | | | <142 | 142–179 | 179–217 | 217–272 | >272 | *P*-value |
|  |  |  | *n*=3,983 | *n*=3,983 | *n*=3,982 | *n*=3,983 | *n*=3,983 |  |
|  | Maternal age, years | | 30.1±5.2 | 31.4±4.9 | 32.0±4.9 | 32.4±4.8 | 32.7±4.8 | <0.0001 |
|  | Body mass index, kg/m^2^ | | 21.7±3.3 | 21.5±3.1 | 21.4±3.0 | 21.4±3.0 | 21.7±3.2 | 0.5 |
|  | Gestational age, weeks | | 39.2±1.7 | 39.2±1.7 | 39.1±1.7 | 39.1±1.7 | 39.1±1.7 | 0.009 |
|  | Current smoking at recruitment, % | | 131 (3.3) | 84 (2.1) | 96 (2.4) | 85 (2.1) | 113 (2.8) | 0.001 |
|  | Current alcohol use at recruitment, % | | 647 (16.4) | 725 (18.2) | 784 (19.7) | 878 (22.1) | 884 (22.2) | 0.03 |
|  | 12 years of education or less, % | | 944 (40.0) | 799 (31.5) | 824 (31.6) | 763 (29.4) | 803 (31.7) | <0.0001 |
|  | Diabetes mellitus (type 1 or 2), % | | 8 (0.3) | 7 (0.3) | 9 (0.3) | 10 (0.4) | 13 (0.5) | 0.9 |
|  | Systemic lupus erythematosus, % | | 1 (0.0) | 4 (0.2) | 5 (0.2) | 6 (0.2) | 3 (0.1) | 0.4 |
|  | Family history of HDP, % | | 53 (2.2) | 79 (3.1) | 76 (2.9) | 80 (3.1) | 87 (3.4) | 0.07 |
|  | Family history of CH | |  |  |  |  |  |  |
|  |  | Paternal history of CH, % | 522 (21.9) | 594 (23.2) | 683 (25.9) | 705 (27.0) | 641 (25.0) | 0.3 |
|  |  | Maternal history of CH, % | 396 (16.6) | 472 (18.4) | 535 (20.3) | 532 (20.4) | 477 (18.6) | 0.09 |
|  |  | Brother's history of CH, % | 14 (0.6) | 19 (0.7) | 30 (1.1) | 25 (1.0) | 27 (1.1) | 0.6 |
|  |  | Sister's history of CH, % | 4 (0.2) | 12 (0.5) | 8 (0.3) | 11 (0.4) | 14 (0.5) | 0.1 |
|  | Parity | |  |  |  |  |  |  |
|  |  | Nulliparous, % | 2,297 (57.8) | 2,047 (51.5) | 1,881 (47.3) | 1,719 (43.2) | 1,599 (40.2) | <0.0001 |
|  |  | Parous with previous HDP, % | 84 (2.1) | 105 (2.6) | 97 (2.4) | 100 (2.5) | 113 (2.8) |  |
|  |  | Parous with no previous HDP, % | 1,591 (40.1) | 1,825 (45.9) | 1,998 (50.3) | 2,157 (54.3) | 2,264 (56.9) |  |
|  |  | Inter-birth interval, years | 3.8±2.6 | 3.6±2.4 | 3.8±2.4 | 3.8±2.5 | 3.8±2.5 | 0.2 |
|  |  | Last delivery gestational age, weeks | 38.9±1.7 | 38.9±1.8 | 39.0±1.9 | 38.9±1.9 | 38.9±1.8 | 0.5 |
|  | Conception by *in vitro* fertilization, % | | 151 (3.8) | 190 (4.8) | 198 (5.0) | 183 (4.6) | 200 (5.0) | 0.04 |
|  | Mean arterial pressure, mmHg | | 81.1±9.8 | 80.8±9.6 | 80.1±9.7 | 79.9±9.7 | 79.5±9.5 | <0.0001 |
|  | Dietary nutrition intake | |  |  |  |  |  |  |
|  |  | Calcium, mg/day | 211.3±106.0 | 339.7±118.3 | 428.9±153.0 | 541.3±196.4 | 911.7±650.3 | <0.0001 |
|  |  | Potassium, mg/day | 997.0±314.9 | 1,532.1±198.1 | 1,903.6±230.9 | 2,357.1±288.2 | 3,614.6±1,501.8 | <0.0001 |
|  |  | Sodium, mg/day | 1,682.2±678.6 | 2,494.2±666.3 | 3,003.2±743.7 | 3,627.6±888.1 | 5,168.0±2,421.0 | <0.0001 |
|  |  | Magnesium, mg/day | 108.8±28.0 | 161.3±10.8 | 197.5±10.8 | 242.2±15.5 | 359.6±135.3 | <0.0001 |
|  |  | Na/K ratio | 1.7±0.6 | 1.6±0.4 | 1.6±0.4 | 1.6±0.4 | 1.5±0.4 | <0.0001 |
|  |  | Energy, kcal/day | 1,047.4±283.8 | 1,388.9±231.8 | 1,597.8±261.8 | 1,847.7±298.5 | 2,507.6±898.6 | <0.0001 |
|  | Subtypes of HDP | |  |  |  |  |  |  |
|  |  | Not affected | 3,451 (86.6) | 3,530 (88.6) | 3,537 (88.8) | 3,581 (89.9) | 3,568 (89.6) | 0.07 |
|  |  | CH | 125 (3.1) | 103 (2.6) | 124 (3.1) | 83 (2.1) | 91 (2.3) |  |
|  |  | Gestational hypertension | 201 (5.0) | 186 (4.7) | 171 (4.3) | 164 (4.1) | 174 (4.4) |  |
|  |  | Pre-eclampsia | 133 (3.3) | 111 (2.8) | 98 (2.5) | 102 (2.6) | 103 (2.6) |  |
|  |  | Superimposed pre-eclampsia | 73 (1.8) | 53 (1.3) | 52 (1.3) | 53 (1.3) | 47 (1.2) |  |
|  |  |  |  |  |  |  |  |  |
| Daily dietary Na/K | | | <1.23 | 1.23–1.45 | 1.45–1.65 | 1.65–1.91 | >1.91 | *P*-value |
|  |  |  | *n*=3,983 | *n*=3,983 | *n*=3,982 | *n*=3,983 | *n*=3,983 |  |
|  | Maternal age, years | | 32.0±5.0 | 32.1±4.8 | 32.1±5.0 | 31.5±4.9 | 30.9±5.1 | <0.0001 |
|  | Body mass index, kg/m^2^ | | 21.5±3.1 | 21.5±3.1 | 21.6±3.1 | 21.5±3.1 | 21.6±3.2 | 0.7 |
|  | Gestational age, weeks | | 39.2±1.6 | 39.1±1.9 | 39.1±1.7 | 39.1±1.7 | 39.2±1.6 | 0.4 |
|  | Current smoking at recruitment, % | | 111 (2.8) | 97 (2.4) | 80 (2.0) | 91 (2.3) | 130 (3.3) | 0.4 |
|  | Current alcohol use at recruitment, % | | 809 (20.4) | 833 (21.0) | 783 (19.7) | 757 (19.0) | 736 (18.6) | 0.6 |
|  | Education ≤ 12 years, % | | 760 (29.5) | 701 (27.7) | 787 (30.6) | 864 (34.0) | 1021 (42.3) | 0.2 |
|  | Diabetes mellitus (type 1 or 2), % | | 15 (0.6) | 8 (0.3) | 10 (0.4) | 10 (0.4) | 4 (0.2) | 0.2 |
|  | Systemic lupus erythematosus, % | | 5 (0.2) | 3 (0.1) | 4 (0.2) | 3 (0.1) | 4 (0.2) | 0.7 |
|  | Family history of HDP, % | | 81 (3.1) | 63 (2.5) | 74 (2.9) | 88 (3.4) | 69 (2.8) | 0.2 |
|  | Family history of CH | |  |  |  |  |  |  |
|  |  | Paternal history of CH, % | 682 (26.2) | 661 (25.8) | 652 (25.2) | 610 (23.8) | 540 (22.1) | 0.8 |
|  |  | Maternal history of CH, % | 509 (19.6) | 476 (18.6) | 505 (19.5) | 517 (20.2) | 405 (16.6) | 0.4 |
|  |  | Brother's history of CH, % | 28 (1.1) | 25 (1.0) | 24 (0.9) | 22 (0.9) | 16 (0.7) | 0.8 |
|  |  | Sister's history of CH, % | 9 (0.3) | 9 (0.4) | 11 (0.4) | 9 (0.4) | 11 (0.5) | >0.99 |
|  | Parity | |  |  |  |  |  |  |
|  |  | Nulliparous, % | 2,030 (51.1) | 1,914 (48.1) | 1,830 (46.0) | 1,842 (46.4) | 1,927 (48.5) | 0.005 |
|  |  | Parous with previous HDP, % | 115 (2.9) | 95 (2.4) | 120 (3.0) | 81 (2.0) | 88 (2.2) |  |
|  |  | Parous with no previous HDP, % | 1,828 (46.0) | 1,969 (49.5) | 2,026 (51.0) | 2,051 (51.6) | 1,961 (49.3) |  |
|  |  | Inter-birth interval, years | 3.8±2.5 | 3.8±2.5 | 3.7±2.5 | 3.7±2.4 | 3.8±2.5 | 0.3 |
|  |  | Last delivery gestational age, weeks | 38.9±1.9 | 38.9±1.9 | 38.9±1.8 | 39.0±1.7 | 39.0±1.8 | 0.04 |
|  | Conception by *in vitro* fertilization, % | | 209 (5.3) | 210 (5.3) | 198 (5.0) | 161 (4.0) | 144 (3.6) | >0.99 |
|  | Mean arterial pressure, mmHg | | 80.1±10.0 | 80.5±9.6 | 80.1±9.6 | 80.4±9.6 | 80.3±9.6 | 0.5 |
|  | Dietary nutrition intake | |  |  |  |  |  |  |
|  |  | Calcium, mg/day | 688.9±649.9 | 510.9±350.1 | 463.6±279.5 | 418.1±249.7 | 351.5±209.9 | <0.0001 |
|  |  | Potassium, mg/day | 2,416.1±1,446.5 | 2,190.0±1,124.5 | 2,091.9±966.0 | 1,966.3±1,013.5 | 1,740.2±946.9 | <0.0001 |
|  |  | Sodium, mg/day | 2,480.0±1,413.8 | 2,947.9±1,509.2 | 3,239.9±1,498.8 | 3,479.2±1,808.8 | 3,828.3±2,038.4 | <0.0001 |
|  |  | Magnesium, mg/day | 237.9±129.4 | 221.8±107.3 | 215.1±90.5 | 205.7±98.4 | 188.9±90.8 | <0.0001 |
|  |  | Na/K ratio | 1.0±0.2 | 1.3±0.1 | 1.5±0.1 | 1.8±0.1 | 2.3±0.4 | <0.0001 |
|  |  | Energy, kcal/day | 1,728.5±758.1 | 1,691.2±643.7 | 1,689.5±595.9 | 1,672.8±668.7 | 1,607.4±710.8 | <0.0001 |
|  | Subtypes of HDP | |  |  |  |  |  |  |
|  |  | Not affected | 3,549 (89.1) | 3,534 (88.7) | 3,558 (89.4) | 3,498 (87.8) | 3,528 (88.6) | 0.3 |
|  |  | CH | 124 (3.1) | 104 (2.6) | 87 (2.2) | 110 (2.8) | 101 (2.5) |  |
|  |  | Gestational hypertension | 152 (3.8) | 168 (4.2) | 183 (4.6) | 197 (4.9) | 196 (4.9) |  |
|  |  | Pre-eclampsia | 97 (2.4) | 119 (3.0) | 98 (2.5) | 123 (3.1) | 110 (2.8) |  |
|  |  | Superimposed pre-eclampsia | 61 (1.5) | 58 (1.5) | 56 (1.4) | 55 (1.4) | 48 (1.2) |  |
|  |  |  |  |  |  |  |  |  |
| Daily dietary energy intake, kcal | | | <1,215 | 1,215–1,461 | 1,461–1,708 | 1,708–2,053 | >2,053 | *P*-value |
|  |  |  | *n*=3,983 | *n*=3,983 | *n*=3,982 | *n*=3,983 | *n*=3,983 |  |
|  | Maternal age, years | | 30.8±5.2 | 31.7±5.0 | 31.8±4.8 | 32.1±4.9 | 32.1±5.0 | <0.0001 |
|  | Body mass index, kg/m^2^ | | 21.6±3.2 | 21.5±3.1 | 21.4±3.0 | 21.5±3.1 | 21.7±3.2 | 0.1 |
|  | Gestational age, weeks | | 39.2±1.6 | 39.2±1.8 | 39.2±1.7 | 39.1±1.8 | 39.1±1.6 | 0.003 |
|  | Current smoking at recruitment, % | | 119 (3.0) | 78 (2.0) | 81 (2.0) | 102 (2.6) | 129 (3.3) | 0.004 |
|  | Current alcohol use at recruitment, % | | 629 (15.9) | 679 (17.1) | 798 (20.1) | 873 (22.0) | 939 (23.6) | 0.2 |
|  | Education ≤ 12 years, % | | 891 (36.6) | 835 (32.5) | 798 (30.5) | 789 (30.7) | 820 (33.5) | 0.002 |
|  | Diabetes mellitus (type 1 or 2), % | | 10 (0.4) | 8 (0.3) | 6 (0.2) | 12 (0.5) | 11 (0.4) | 0.7 |
|  | Systemic lupus erythematosus, % | | 2 (0.1) | 3 (0.1) | 4 (0.2) | 6 (0.2) | 4 (0.2) | >0.99 |
|  | Family history of HDP, % | | 68 (2.8) | 64 (2.5) | 73 (2.8) | 85 (3.3) | 85 (3.4) | 0.6 |
|  | Family history of CH | |  |  |  |  |  |  |
|  |  | Paternal history of CH, % | 558 (22.7) | 608 (23.4) | 678 (25.8) | 667 (25.7) | 634 (25.7) | 0.6 |
|  |  | Maternal history of CH, % | 427 (17.4) | 477 (18.4) | 510 (19.4) | 516 (19.9) | 482 (19.5) | 0.4 |
|  |  | Brother's history of CH, % | 17 (0.7) | 19 (0.7) | 22 (0.8) | 31 (1.2) | 26 (1.1) | >0.99 |
|  |  | Sister's history of CH, % | 9 (0.4) | 2 (0.1) | 11 (0.4) | 15 (0.6) | 12 (0.5) | 0.06 |
|  | Parity | |  |  |  |  |  |  |
|  |  | Nulliparous, % | 2,245 (56.5) | 2,000 (50.3) | 1,888 (47.5) | 1,728 (43.4) | 1,682 (42.3) | <0.0001 |
|  |  | Parous with previous HDP, % | 89 (2.2) | 101 (2.5) | 82 (2.1) | 113 (2.8) | 114 (2.9) |  |
|  |  | Parous with no previous HDP, % | 1,640 (41.3) | 1,872 (47.1) | 2,004 (50.4) | 2,140 (53.8) | 2,179 (54.8) |  |
|  |  | Inter-birth interval, years | 3.8±2.6 | 3.7±2.5 | 3.7±2.3 | 3.7±2.4 | 3.9±2.6 | 0.1 |
|  |  | Last delivery gestational age, weeks | 38.9±1.7 | 38.9±1.8 | 39.0±1.7 | 38.9±1.9 | 38.8±1.9 | 0.2 |
|  | Conception by *in vitro* fertilization, % | | 192 (4.8) | 194 (4.9) | 182 (4.6) | 175 (4.4) | 179 (4.5) | 0.96 |
|  | Mean arterial pressure, mmHg | | 81.0±9.9 | 80.6±9.7 | 80.1±9.4 | 79.8±9.5 | 79.9±9.8 | <0.0001 |
|  | Dietary nutrition intake | |  |  |  |  |  |  |
|  |  | Calcium, mg/day | 241.8±134.8 | 355.2±148.2 | 437.4±182.8 | 528.4±226.6 | 870.1±664.1 | <0.0001 |
|  |  | Potassium, mg/day | 1,110.4±431.3 | 1,594.2±381.7 | 1,935.9±452.8 | 2,329.9±530.7 | 3,433.9±1,598.6 | <0.0001 |
|  |  | Sodium, mg/day | 1,779.3±771.7 | 2,508.1±749.2 | 3,022.3±831.7 | 3,593.7±967.3 | 5,071.9±2,456.0 | <0.0001 |
|  |  | Magnesium, mg/day | 118.7±38.8 | 168.1±32.6 | 200.9±38.2 | 239.7±46.1 | 342.0±145.4 | <0.0001 |
|  |  | Na/K ratio | 1.6±0.6 | 1.6±0.4 | 1.6±0.4 | 1.6±0.4 | 1.5±0.4 | <0.0001 |
|  |  | Energy, kcal/day | 977.3±209.2 | 1,343.3±70.5 | 1,581.9±71.6 | 1,863.7±97.8 | 2,623.2±836.9 | <0.0001 |
|  | HDP subtypes | |  |  |  |  |  |  |
|  |  | Not affected | 3,457 (86.8) | 3,537 (88.8) | 3,610 (90.7) | 3,545 (89.0) | 3,518 (88.3) | 0.02 |
|  |  | CH | 130 (3.3) | 114 (2.9) | 85 (2.1) | 90 (2.3) | 107 (2.7) |  |
|  |  | Gestational hypertension | 210 (5.3) | 156 (3.9) | 156 (3.9) | 192 (4.8) | 182 (4.6) |  |
|  |  | Pre-eclampsia | 113 (2.8) | 116 (2.9) | 97 (2.4) | 103 (2.6) | 118 (3.0) |  |
|  |  | Superimposed pre-eclampsia | 73 (1.8) | 60 (1.5) | 34 (0.9) | 53 (1.3) | 58 (1.5) |  |

Data are expressed as means (standard deviation) for continuous variables and *n* (%) for categorical variables.
*P* values were evaluated by analysis of variance for continuous variables and the chi-squared or Fisher's exact test for categorical variables.
HDP, hypertensive disorders of pregnancy; CH, chronic hypertension. Na/K ratio, sodium-to-potassium intake ratio;
Dietary intakes are shown without adjustment for energy intake.
