# Supplementary material for: Dietary calcium intake was related to the onset of pre‐eclampsia: The TMM BirThree Cohort Study
Source: J Clin Hypertens (Greenwich). 2022 Dec 28;25(1):61–70. doi: 10.1111/jch.14606 (PMC9832228; doi:10.1111/jch.14606)
Supplement: Supplementary file 3 — Supporting Information [file JCH-25-61-s003.docx]

**Supplemental Table 2.** AIC among prediction models with various variable transformations

|  | Linear | Quadratic | Broken-stick |
| --- | --- | --- | --- |
| Calcium | 3,787.3 | 3,786.4 | 3,777.3* |
| Potassium | 3,787.4 | 3,787.1 | 3,783.2* |
| Sodium | 3,786.4 | 3,785.8 | 3,782.4* |
| Magnesium | 3,787.7 | 3,787.6 | 3,785.6* |
| Na/K ratio | 3,788.3 | 3,788.3* | - |
| Energy | 3,787.9 | 3,787.8* | - |
| Energy-adjusted calcium | 3,788.2 | 3,788.2 | 3,787.4* |
| Energy-adjusted potassium | 3,788.5 | 3,788.3* | 3,788.9 |
| Energy-adjusted sodium | 3,787.6 | 3,787.6* | 3,788.3 |
| Energy-adjusted magnesium | 3,788.8 | 3,788.5* | 3,788.8 |
| Energy-adjusted Na/K ratio | 3,788.2 | 3,788.2* | - |

AIC, Akaike's Information Criterion; Na/K ratio, sodium-to-potassium intake ratio.
* represents the model with the lowest AIC for that electrolyte.
AIC was calculated in the competing risk model without adjustment for basic characteristics.
We presumed a broken-stick model with daily break-points for calcium (600 mg), potassium (2,000 mg), sodium (3,000 mg), magnesium (200 mg), energy-adjusted calcium (600 mg), energy-adjusted potassium (2,000 mg), energy-adjusted sodium (3,000 mg), and energy-adjusted magnesium (200 mg) based on the scatter plot (Figure 1), but not for Na/K ratio, energy, energy-adjusted Na/K ratio.
